# Time and rate dependent synaptic learning in neuro-mimicking resistive memories

**DOI:** 10.1038/s41598-019-51700-0

**Published:** 2019-10-28

**Authors:** Taimur Ahmed, Sumeet Walia, Edwin L. H. Mayes, Rajesh Ramanathan, Vipul Bansal, Madhu Bhaskaran, Sharath Sriram, Omid Kavehei

**Affiliations:** 1 Functional Materials and Microsystems Research Group and Micro Nano Research Facility, RMIT University, Melbourne, VIC 3001 Australia; 2 RMIT Microscopy and Microanalysis Facility, RMIT University, Melbourne, VIC 3001 Australia; 3 Sir Ian Potter NanoBioSensing Facility, NanoBiotechnology Research Laboratory, School of Science, RMIT University, Melbourne, VIC 3001 Australia; 4 Present Address: Faculty of Engineering, The University of Sydney, NWS 2006 Sydney, Australia

**Keywords:** Electrical and electronic engineering, Electronic devices, Information storage

## Abstract

Memristors have demonstrated immense potential as building blocks in future adaptive neuromorphic architectures. Recently, there has been focus on emulating specific synaptic functions of the mammalian nervous system by either tailoring the functional oxides or engineering the external programming hardware. However, high device-to-device variability in memristors induced by the electroforming process and complicated programming hardware are among the key challenges that hinder achieving biomimetic neuromorphic networks. Here, a simple hybrid complementary metal oxide semiconductor (CMOS)-memristor approach is reported to implement different synaptic learning rules by utilizing a CMOS-compatible memristor based on oxygen-deficient SrTiO_3*-x*_ (STO_*x*_). The potential of such hybrid CMOS-memristor approach is demonstrated by successfully imitating time-dependent (pair and triplet spike-time-dependent-plasticity) and rate-dependent (Bienenstosk-Cooper-Munro) synaptic learning rules. Experimental results are benchmarked against *in-vitro* measurements from hippocampal and visual cortices with good agreement. The scalability of synaptic devices and their programming through a CMOS drive circuitry elaborates the potential of such an approach in realizing adaptive neuromorphic computation and networks.

## Introduction

The functionality of a brain is attributed to the activity-dependent synaptic weight change, enabling principal cognitive functions^1^. Although the underlying precise biological mechanism of the synaptic functionality is still under debate^2^, it is well established that *in vivo* neurons follow the anti-Hebbian synaptic learning through spike-time-dependent-plasticity (STDP)^3–7^. In order to mimic the biological synaptic learning, conventional CMOS circuits have been employed^8,9^, but their limited scalability poses challenge to achieve the efficiency^10^ and density (~10^11^ neurons and ~10^15^ synapses compact in volume of ~1130-1260 cm^3^)^11^ of the human brain.

Nanoscale memristors have attracted attention as potential artificial synapses due to their similar activity-dependent nonlinear conductance modulation^12–15^. However, memristors require integration with the driving CMOS subsystems to successfully execute the memory/computation operations and emulate synaptic functions. To date, several hybrid CMOS-memristor architectures have been reported to achieve high density memory systems and neuromorphic computing paradigm^16–18^. But CMOS circuitry specifically designed for a particular memristor type, inexorable electroforming process causing a high device-to-device variability and associated stochastic nature of resistive switching are hampering the realization of efficient neuromorphic networks^15,16,19^. A hybrid architecture implementing a simple dynamic CMOS circuitry to comply with any type of memristors and an electroforming-free memristor would enable the imitation of versatile neuromorphic functions.

In this study, we exploit characteristics of STO_*x*_ memristors^20–23^ and flexible design of a CMOS drive circuit to imitate different time and rate dependent synaptic functions. Over the recent few years, a variety of synaptic functions including short- and long-term memory, paired-pulse facilitation and pair-based STDP (*p-*STDP) have been implemented on different types of memories^11,24–28^. However, experimental demonstrations of higher order time-dependent plasticity such as triple-STDP (*t*-STDP) and quadruplet-STDP (*q*-STDP), and Bienenstock-Cooper-Munro (BCM) synaptic modifications (well known to exist in biological synapses)^29–31^ are not extensively explored in artificial synaptic devices. Though the classical *p*-STDP models helped to establish a fundamental understanding of the Hebbian synaptic plasticity in several neural systems but it is not sufficient to accurately model all biological experimental results produced by multiple (triplet and quadruplet) spikes^29,32^. This can be associated with deficiencies in the classical *p*-STDP model, such as excluding non-linear integration of spike pairs and their repetition frequency to quantify the synaptic modification^30,32^. This infers that the classical *p*-STDP model cannot elicit the BCM synaptic learning rule, which is regarded as a possible explanation of experience-dependent synaptic plasticity^30^. On the other hand, the *t*-STDP model is believed to be comprehensive enough to explain the experimental results produced by multiple spikes in biological neural systems. As such, the implementation of the *t*-STDP rule on STO_*x*_ synaptic devices can highlight the capability of these artificial synapses to mimic the biological synaptic functionalities. We acknowledge that a few memristive models and circuits have recently been proposed to reproduce these synaptic learning rules^33–35^. However, an experimental emulation of these essential biological learning rules will signify the potential of memristors for future neuromorphic computation.

Furthermore, the available literature reports either extensive peripheral circuitry to generate suitable pre- and post-synaptic spike shapes (similar to the biological action potentials) or special circuits designed for a specific type of memristive system^16,36^. Additionally, the direct interfacing between CMOS drive circuitry and memristive devices/array can expose them to CMOS circuit non-ideality^37^. Herein, we utilize a well-established CMOS circuit, called forward body biasing^38–41^, in combination with a time-to-digital converter to implement not only time-dependent synaptic rules but also demonstrate the potential of implementing a wide variety of synaptic learning rules.

## Results and Discussion

### Switching characteristics of STO_*x*_ synaptic devices

**Figure 1:**
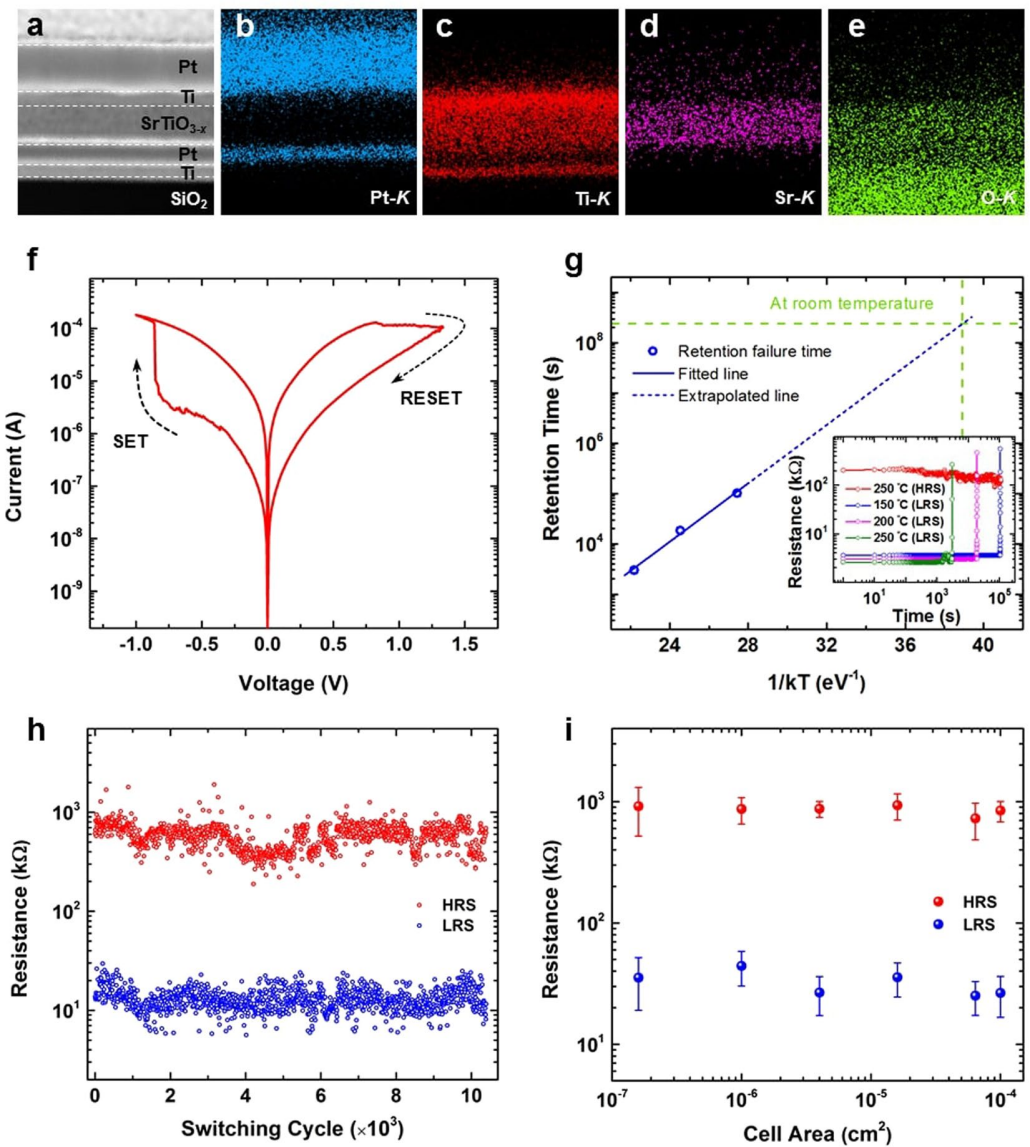
The STO_*x*_ synaptic devices. (**a**) The cross-sectional scanning transmission electron microscope micrograph of a pristine STO_*x*_ synaptic device. Scale bar 50 nm. (**b**) The energy-dispersive X-ray spectroscopic elemental maps of (**b**) Pt, (c) Ti, (**d**) Sr and (**e**) O. (**f**) The *I–V* characteristic sweep of a 10 × 10 µm^2^ STO_*x*_ MIM device. (**g**) The retention time *vs*. 1/kT plot to evaluate the state stability of the STO_*x*_ devices. The inset shows retention of LRS and HRS at different elevated temperatures ranging from 150 to 250 °C. (**h**) Endurance of the devices, where *V*_RESET_ of −1.6 V, *V*_SET_ of +1.4 V and *V*_READ_ of +0.1 V are applied as a train of short pulses. (**i**) The dependence of HRS and LRS on the active cell area.

The metal-insulator-metal (MIM) configuration of the synaptic devices is assessed by the cross-sectional scanning transmission electron microscope (STEM, Fig. 1a) and energy-dispersive X-ray spectroscopic (EDS) elemental maps (Fig. 1b–e). The stoichiometric analysis of STO_*x*_ thin films shows that the sputtered films are oxygen-deficient which indicates presences of oxygen vacancies in the as-grown STO_*x*_ switching layer (Supplementary Information, Fig. S1). Figure 1f shows a clockwise bipolar switching behavior of the STO_*x*_ synaptic devices. A negative quasi-static voltage sweep with amplitude of -1 V (as *V*_SET_) switches the MIM device from its high resistance state (HRS) to a low resistance state (LRS). While an opposite polarity quasi-static voltage sweep with an amplitude of + 1.3 V (as *V*_RESET_) switches the device to its HRS. This behavior *i*.*e*., RESET on positive bias and SET on negative bias is typical for STO-based memristors^21–23,42^. The as-fabricated MIM devices are in their high resistive state (HRS) as the measured pristine resistances are close to the normal variance of HRS achieved during subsequent cyclic switching (Supplementary Information, Fig. S2). However, the pristine state resistances are cell-area dependent. Furthermore, the statistical analysis of the as-fabricated devices shows that the average SET and RESET voltages during the first *I–V* sweeps are also area dependent (Supplementary Information, Fig. S2). The top Ti layer at Ti/STO_*x*_ interface plays an important role in defining the switching characteristics of STO_*x*_ resistive memories. In as-fabricated devices under zero bias condition, Ti layer partially oxidizes to sub-stoichiometric oxide (such as Ti_2_O_3_)^43,44^ due to the interfacial oxygen diffusion and Ti‒O bonding between Ti and STO_*x*_ oxygen ions, at the vicinity of underlying STO_x_ (discussed in cross-sectional analysis below). This introduces an additional switching layer at the top interface (*i*.*e*., Ti/Ti_2_O_3_/STO_*x*_) which is electrochemically different to oxygen deficient STO_*x*_ and causes a change in mobility and formation energies of oxygen-vacancies in Ti_2_O_3_/STO_*x*_ heterostructure. As such, the STO_*x*_ synaptic devices do not require explicit electroforming and exhibit resistive switching after a low conditioning voltage sweep (close to the subsequent SET voltage sweeps). This can be associated with (i) the presence of as-grown oxygen vacancies in STO_*x*_ thin films^45,46^ (revealed by X-ray photoelectron spectroscopy and photoluminescence spectra, Section S1 of Supplementary Information) and (ii) it is possible that during the first SET sweep Joule heating may induce additional oxygen vacancies in the MIM structure, according to oxygen exchange reaction^47^. As such, the increasing concentration of oxygen vacancies reduces their migration distance and consequently the electrical energy required to form a conductive filamentary path. Further evidence and cross-sectional characterization of the filamentary path is given in the following sections.

To evaluate the reliability of the STO_*x*_ MIM devices, the resistive states are measured at elevated temperatures ranging from 150 to 250 °C, as shown in the inset of Fig. 1g. The retention of HRS measured for 30 hours at 250 °C shows no failure, indicating high stability of HRS. However, retention characteristics of LRS are temperature-sensitive. This high temperature LRS retention failure can be associated with the thermally-assisted reduction in the concentration of oxygen vacancies in the nano-filament and eventually leading its rupture^48^. The LRS retention failure time at different temperatures (where resistance jumps higher than the HRS) is plotted in an Arrhenius plot, as shown in Fig. 1g, to calculate the oxygen vacancy migration activation energy and estimate the retention characteristics of the STO_*x*_ memristors. The extrapolation of the fitting line in Fig. 1g estimates the retention time of *ca*. 7.6 years at room temperature. Even though, this retention is suitable for memory and neuromorphic applications, it may be further improved by preventing the re-oxidation of STO_*x*_ oxide layer through inserting a thin film exhibiting slow oxygen diffusion coefficient, such as Al_2_O_3_^49^. On the other hand, activation energy of *ca*. 0.29 eV is extracted from the linear fitting of the experimental data. Such a low LRS activation energy, as compared to the other oxide systems^48,50,51^ (*e*.*g*., 1.0–1.6 eV reported for amorphous Al_2_O_3_, amorphous Ta_2_O_5_, amorphous Nb_2_O_5_, and TiO_2_), suggests a hopping conduction mechanism in our STO_*x*_ MIM devices^52^. This hopping conduction refers to the electronic transport through localized states, where these states are provided by the oxygen vacancies in the nano-filament.

To evaluate the switching repeatability of the STO_*x*_ MIM devices (Fig. 1h), short pulses of 1 μs duration and amplitude of −1.4 V and +1.6 V are applied for SET and RESET operations, respectively. READ pulses with amplitude of +0.1 V and duration of 200 ns are used to measure the SET/RESET currents. The effect of pulse width on the switching performance is also evaluated (Supplementary Information, Fig. S4). The endurance characteristics for more than 10^4^ switching cycles (Fig. 1h) indicate that the synaptic devices exhibit repeatable bipolar switching behaviour. Typically in transition metal oxides, the bipolar resistive switching behavior is attributed to the inhomogeneous conduction mechanisms through the localized filamentary pathways and associated redox processes^47,53–55^. As such, the resistance states (*i*.*e*., HRS and LRS) are expected to be independent of the lateral dimensions of the MIM devices. Figure 1i reveals no appreciable area-dependency in our STO_*x*_ devices for either resistance state. This further supports our earlier statement regarding the formation of conductive filamentary pathway in the MIM devices.

### Visualising filamentary switching in resistive states

**Figure 2:**
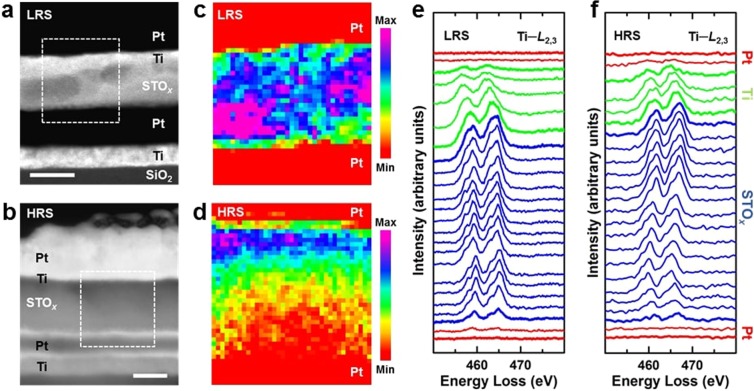
Microstructural and compositional analyses of the STO_*x*_ synaptic devices. (**a**) STEM cross-section of a switching device in its LRS. Scale bar 20 nm. (**b**) STEM cross-section of a switching device in its HRS. Scale bar 20 nm. (**c**) The EELS O*–K* edge area map of the enclosed region of interest in (**a**). The colour bar shows the relative oxygen content. (**d**) The EELS O*–K* edge area map of the enclosed region of interest in (**b**). The colour bar shows the relative oxygen content. (**e**) The EELS Ti*–L*_2,3_ edge profiles along a line scan across the ROI in (**a**). (**f**) The EELS Ti*–L*_2,3_ edge profiles along a line scan across the ROI in (**b**).

The physical structure of the STO_*x*_ synaptic devices and their compositional analysis is characterized by cross-sectional transmission electron microscopy (TEM). Electron energy loss spectroscopy (EELS) is used to assess the distribution of oxygen content in the MIM devices. The cross-sectional micrograph and corresponding EELS spectra of a pristine MIM device reveal an amorphous microstructure of the STO_*x*_ layer and a partial oxidation of the top Ti layer at the Ti/STO_*x*_ interface (Supplementary Information, Fig. S5). The amorphous and oxygen-deficient structure of STO_*x*_ layer is attributed to the room temperature sputtering in a pure argon environment. Also, the partial oxidation of the top Ti layer to sub-oxide at Ti/STO_*x*_ interface can be associated with the interfacial oxygen diffusion and Ti‒O bonding between Ti and STO_*x*_ oxygen ions^44,56,57^. Fig. 2a,b shows scanning TEM (STEM) images of the switching STO_*x*_ memristive devices in their LRS and HRS, respectively. High contrast regions are observed in the STO_*x*_ layers and along the top Ti/STO_*x*_ interfaces which indicate the applied electric field induced compositional changes in the STO_*x*_ layers. To analyse the state-dependent composition of the STO_*x*_ layers, region of interests (ROIs) are selected across the lamellae, highlighted in Fig. 2a,b. The EELS O–K edge area maps (Fig. 2c,d) show the relative distribution of oxygen content in ROIs where the area maps are generated by taking the O–K edge intensities of the collected spectra (at each pixel) after pre-edge background subtraction. The O–K edge area map of the device in LRS (Fig. 2c) reveals the presence of an extending oxygen-deficient region between top and bottom Pt electrodes. This indicates a localized accumulation of oxygen vacancies and formation of conductive filamentary path across the MIM structure^42^. On the other hand, the O–K edge area map of the device in HRS (Fig. 2d) shows higher concentration of oxygen vacancies at the vicinity of bottom Pt electrode which indicates a ruptured filamentary path.

The formation of each oxygen vacancy in STO introduces two electrons into the Ti 3*d* orbital, and the resulting change in the Ti valence can be observed in the EELS Ti*–L*_2,3_ edge profile^58,59^. Figure 2e,f show the background corrected Ti*–L*_2,3_ spectra acquired along the EELS cross-sectional line scans passing over the ROIs indicated in Fig. 2a,b, respectively. The Ti*–L*_2,3_ fine structures gradually evolve in their intensity and position (from top Pt/Ti interface to the bottom Pt electrode) as clearly observed in both LRS and HRS. Due to resolution limitation, we evaluate the Ti*–L*_2,3_ edge profiles to qualitatively analyse the electronic structure of the ROIs. The broad and relatively low intensity peaks at top Pt/Ti interface indicate the presence of mixed Ti^2+^ and Ti^3+^ oxidation states which further highlights the oxidation of Ti layer^44,56,58,60^. At Ti/STO_*x*_ interfacial region and in the STO_*x*_ layer, the crystal-field splitting of Ti*–L*_3_ and Ti*–L*_2_ peaks (into *t*_2g_ and *e*_g_ peaks) and their shift can be attributed to the presence of Ti^3+^ and Ti^4+^ oxidation states^61–63^. It is well known that in transition metal oxide based resistive memories the resistive switching is attributed to the redox reactions and associated valence change in the transition metal cations, such as Ti in STO^47,64^. As such, the cross-sectional TEM analysis shows that the bipolar resistive switching in our STO_*x*_ MIM devices is of filamentary nature where formation and rupture of extended oxygen-deficient regions and associated change in Ti valance result in LRS and HRS, respectively.

### Implementation of synaptic functions

**Figure 3:**
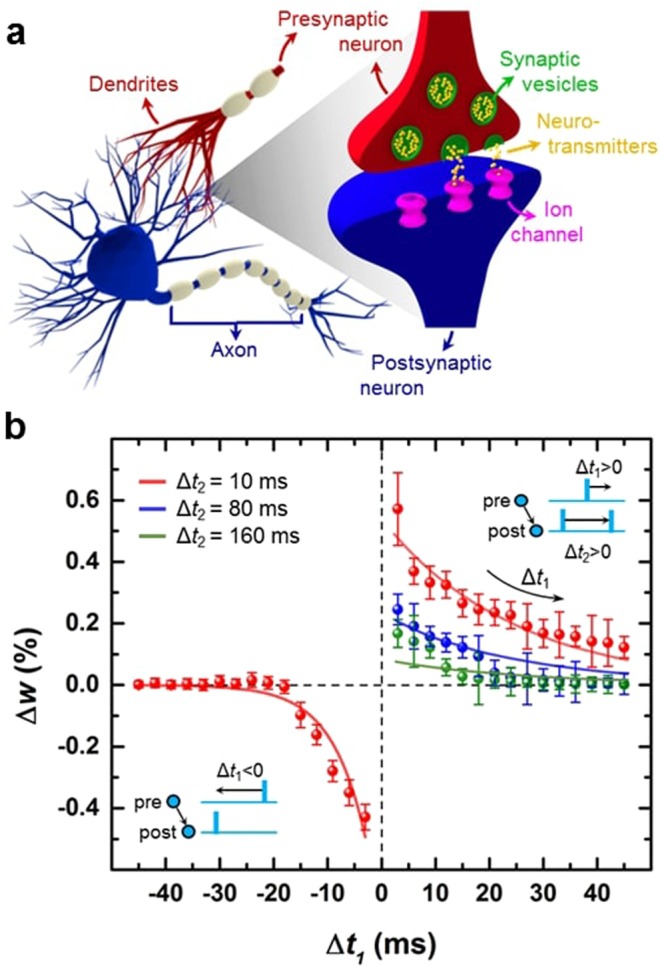
Triplet-based STDP implemented on STO_*x*_ synaptic devices. (**a**) An illustration of two biological neurons connecting *via* synapses. (**b**) Artificial implementation of STDP learning rules using STO_*x*_ synaptic devices. Each data point and its deviation from mean (represented by bars) are collected by applying 100 cycles of identical pulses, where each cycle contains a RESET (for potentiation experiments) or SET (for depression experiments) pulse.

A typical biological synapse consists of a pre-synaptic neuron and a post-synaptic neuron connected through a synaptic cleft, as schematically illustrated in Fig. 3a. In a memristor-based artificial synapse, the bottom and top electrodes work as neurons and the switching layer acts as a synaptic connection. The electrical conductivity of the device interprets the synaptic weight, while its increase or decrease translates to potentiation or depression, respectively, in response to the applied voltage spikes. Figure 3b shows an experimental implementation of simplified *t*-STDP learning rules using our STO_*x*_ memristive devices (cross-point and 7 × 7 array of devices). The protocols to implement these learning rules are adopted from ref. ^29,30^. Synaptic weight changes reported here (denoted by Δ*w* in Fig. 3b) are extracted by applying voltage pulses of different amplitude but fixed pulse width (100 µs). A proposed programming time-to-digital-to-voltage circuitry (discussed in Section 2.4 below) is simulated to generate the amplitude of voltage pulses corresponding to different spike-timing information (*Δt*_1_ and *Δt*_2_). The capability of this scheme to implement a wide range of learning rules, including *p-*STDP and *t*-STDP, is verified by applying a series of 100 pulses for each voltage amplitude that is chosen by the programming circuitry. The amplitude of applied voltage pulses for the corresponding spike-timing is listed in Table S1 (see Supplementary Information). These experiments demonstrate a simple analog time-multiplexing implementation of the artificial memristive synapses with shared peripheral circuitry.

A simplified *t*-STDP learning rule can be presented as^30^

1 $$\begin{array}{rll}{{\Delta }{w}^{-}({\Delta }{t}_{1})} & = & {-{A}_{1}^{-}{e}^{({\Delta }{t}_{1}/{\tau }_{-})},{\Delta }{t}_{1} < 0}\\{{\Delta }{w}^{+}({\Delta }{t}_{1},{\Delta }{t}_{2})} & = & {{A}_{2}^{+}{e}^{(-{\Delta }{t}_{1}/{\tau }_{+})}{e}^{(-{\Delta }{t}_{2}/{\tau }_{y})},}\,\,\,\,\,\,\,\,{{\Delta }{t}_{1}\ge 0,\,{\Delta }{t}_{2}\ge 0}\end{array}$$

where *Δt*_1_(=*t*^post^ - *t*^pre^) and *Δt*_1_(=*t*^post1^ - *t*^post2^) are time differences. ${A}_{1}^{-}\,$and ${A}_{2}^{+}$ are constant amplitudes of each exponential term in potentiation (*Δw*^+^) and depression (*Δw*^−^) equations. The values of these amplitudes extracted from curve fitting (in Fig. 3b) are ${A}_{1}^{-}=-\,0.70$ and ${A}_{2}^{+}=0.60$. Also, *τ*_+_ and *τ*_−_ are time constants of *Δw*^+^ and *Δw*^−^, respectively, and obtained from the fitting parameters as *τ*_+_ = 8.2 ms and *τ*_−_ = 2.5ms. While the time constant *τ*_*y*_ indicates the exponential correlation between *Δw*^+^ and *Δt*_2_, and extracted as *τ*_*y*_ = 80ms. To reproduce the *t*-STDP window, the values of Δ*t*_2_ are fixed at 10, 80 and 160 ms during the experiments (as shown in Fig. 3b).

The simplified *t*-STDP learning rule^29,30^, suggests that synaptic depression is produced by spiking pair with time interval of *Δt*_1_ (as in classical *p*-STDP rule), while synaptic potentiation takes a triplet of spikes into account. It is worth mentioning that ref.^27^ utilizes two sets of spikes. First set of spikes consists of two presynaptic spikes and one postsynaptic spike with *Δt*_1_ and *Δt*_2_ representing the time differences between the postsynaptic spike and the first and second presynaptic spikes, respectively. Second set of spikes includes two postsynaptic spikes and one presynaptic spike. On the other hand, in ref.^28^ the synaptic depression is response to a pre- and postsynaptic pair, while synaptic potentiation is induced with a set of triple spikes which consists of two postsynaptic spikes and one presynaptic spike. In our case, we consider ‘post–pre–post’ configuration of the triplet spikes for synaptic potentiation, all details can be similarly applied for a ‘pre–post–pre’ configuration. Also, a pair of pre- and post-synaptic spikes for synaptic depression.

**Figure 4:**
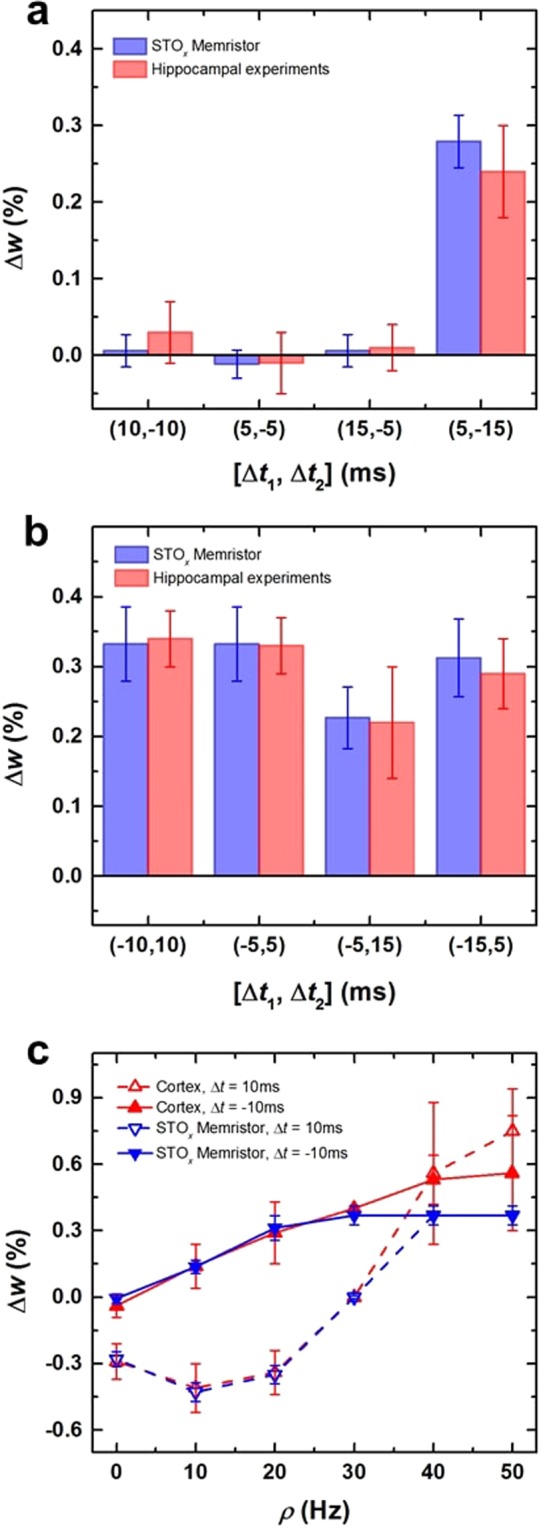
Reproduction analyses of the time- and rate-dependent learning rules. The reproduction of weight change induced by (**a**) pre-post-pre and (**b**) post-pre-post triplet spike patterns. (**c**) The synaptic weight change as a function of spike rate.

In order to demonstrate that our STO_*x*_ synaptic devices are capable of imitating biological synaptic plasticity, we implement the *t*-STDP model (Eq. 1) by following the experimental protocols reported by Pfister and Gerstner^29^. We compare the results with the electrophysiological experiments performed in hippocampal culture^65^ and visual cortex^31^ (Fig. 4). Two different triplet spiking patterns, namely ‘post–pre–post’ (*i*.*e*., 1-pre–2-post) and ‘pre–post–pre’ (*i*.*e*., 2-pre–1-post), are used in hippocampal culture experiments^65^. Each spiking pattern consists of 60 triplet of spikes and are repeated at a rate of 1 Hz. The weight change as a function of timing difference between pre- and post-synaptic spikes in both triplet patterns is graphically presented in Fig. 4a,b. The best fit is calculated by a normalized mean-square error function (*E*) represented as^29^,

2 $$E=\frac{1}{P}\mathop{\sum }\limits_{i=1}^{P}\,{(\frac{\Delta {w}_{i}^{exp}-\Delta {w}_{i}^{mem}}{{\sigma }_{i}})}^{2}$$

where *P*, ${\Delta }{W}_{i}^{exp}$, $\varDelta {W}_{i}^{mem}$ and *σ*_*i*_ are the number of data points in a dataset, mean weight change (in electrophysiological and *a*-STO_*x*_ memristor experiments) and the standard error mean (SEM) of $\varDelta {W}_{i}^{exp}$ for a given data po*i*nt *i*, respectively. In the hippocampal culture, 13 data points are used, which includes 2 pairing and 3 quadruplet data points. To compare our experimental results with hippocampal culture, we use only 8 triplet data points, 4 for 2-pre–1-post and 4 for 1-pre–2-post triplet spiking patterns. Also, we minimize the function *E* (given in Eq. 2) which represents the error between our memristor experimental results and hippocampal culture (Fig. 4a,b). Conventionally, parameters of the CMOS drive circuit are tuned to achieve the best match between the mathematical *t*-STDP and hippocampal culture data^33,34^. However, we minimize the error *via* one-to-one mapping of the weight changes (*Δw*) to the appropriate voltage levels applied to the artificial synapses (STO_*x*_ memristors). Furthermore, this mapping is carried out by extracting timing information using a time-to-digital and then digital-to-voltage conversion in the CMOS drive circuitry (discussed in Section 2.4 below and Section S5 in Supplementary Information). As such, the error is minimized by creating and changing a digital look-up table that maps incoming spike-timing information to a 6-bit digital code. The weight change corresponding to the both triplet pairing configurations is listed in Table S2 (see Supplementary Information). There is <10% error between our memristor data and hippocampal culture data which is comparatively smaller than previously achieved (35%) by mathematical *t*-STDP models^33,34^.

Figure 4c shows the implementation of BCM learning rule where the synaptic weight changes as a function of the given frequency, ρ. The comparison of our experimental results with the visual cortex data set (Fig. 4c) shows that STO_*x*_ memristors closely follow the BCM behavior for ρ ≤ 30 Hz, while for high frequencies our experimental results are within the variation limits of visual cortex data set. As observed in the *I–V* characteristics of the STO_*x*_ memristors, SET process exhibits a digital-like behavior while RESET is an analog-like switching behavior offering comparatively more intermediate stable-states. As such, achieving a high dynamic range of weight change for *Δw*^+^ is more challenging than *Δw*^−^, also observed in Fig. 3b,c. The values of synaptic weight change corresponding to different frequencies are listed in Table S3 (see Supplementary Information). This indicates that similar to time-dependent learning rules (*i*.*e*., *p*-STDP and *t*-STDP); rate dependent learning rules such as BCM rule can also be implemented by our STO_*x*_ synaptic devices.

### CMOS drive circuitry

Generally, to implement STDP with memristors an approach where pre- and post-spikes are superposed to induce synaptic weight change in memristors is adopted. In such approaches input voltage signals (spikes) are directly applied to the devices which can *expose* them to CMOS circuit non-idealities^35,66^. Since the memristive devices response to shape and frequency of an incoming spikes, time-modulated amplitude of superposed spikes or similar techniques are also adopted^66,67^. However, in this work the relative weight change is achieved by a fully digital spike processing unit (time-to-digital-to-voltage circuit, discussed below) which offers a higher level of multiplexing and lower complexity of the overall drive circuitry. Furthermore, through a fully digital spike processing module (time-to-digital-to-voltage) we distinguish from the conventional approaches of placing memristors in the pathway of signals.

**Figure 5:**
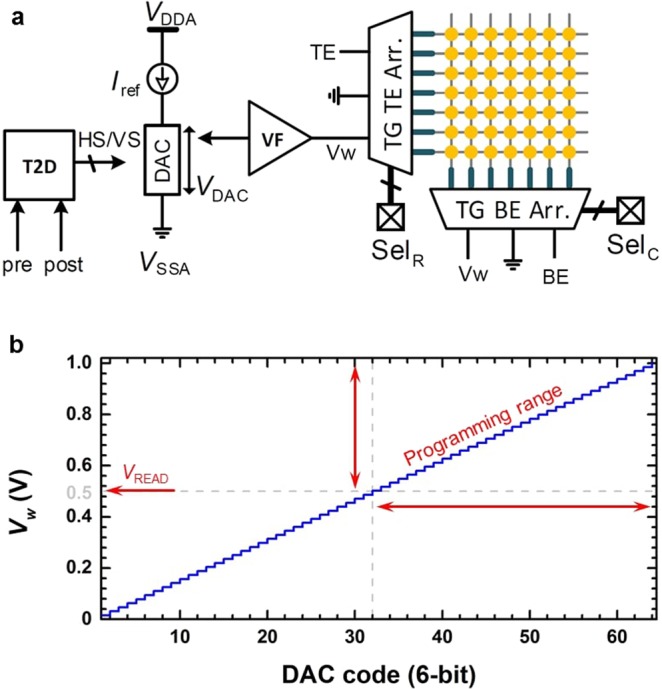
The CMOS drive circuitry. (**a**) A schematic of the proposed CMOS drive circuit which converts difference in input spike-timing into voltage amplitudes to modify the synaptic weight of a target memristor in the array. (**b**) Simulated resolution of the DAC circuitry to generate the weight changing voltage, *i*.*e*., *V*_*w*_.

Figure 5a shows a schematic of the proposed CMOS drive circuit which is a modification of the body-bias generator^39,40^, and converts differences in input spike-timing to voltage amplitudes. The time-to-digital (T2D) module is responsible for the pre- and post-synaptic event digitization and includes a timing control unit and a decoder (see Section S5 in Supplementary Information). The timing control unit is a fully digital unit that receives pre- and post-spikes and generates a binary code according to the timing intervals and works based on a number of counters that are triggered and stopped with spikes. It can be configured to implement multiple protocols and *Δt* detections^38^.

As depicted in Fig. 5a, a finely tuned voltage (*Vw*) is generated to modify the weight of a memristor and is connected to a memristor array *via* a voltage follower (VF) and an array of transmission gates (TGs) that are connected to the top-electrodes (TEs) and bottom-electrodes (BEs) of the memristor array. It is worth mentioning that we focus on the design of peripheral circuitry for memristive artificial synapses, while modifications in the neuron designs may also be necessary to consider for online-learning aspects of the synaptic rules. Each device in the array is individually accessible *via* an addressable top-electrode (TE) and bottom-electrode (BE) connections. Selections are mandated externally and partially include some internal data. These selection signals are represented with two digital vectors for rows (R) and columns (C), *i*.*e*., *Sel*_*R*_ and *Sel*_*C*_ respectively, in Fig. 5a. Note that *I*_*ref*_ is a constant reference current that is supplied through a digital to analog converter (DAC), while *V*_*DDA*_ and *V*_*SSA*_ represent analog voltage supply and ground of the drive circuit, respectively. The proposed drive circuitry disconnects timing scales from the voltage level generation. Also, the T2D module is fully programmable and can map any spike-timing to any binary code which can be translated to the corresponding voltage amplitude, *via* the DAC. It is worth mentioning that addressing an individual device within an array requires additional devices (such as selectors) to protect each device from random programming through parasitic current paths. Also, we are using STO_*x*_ memristors as a multi-state memory device, as reported in our previous work^22^. Therefore, unless an initialization is required, there is no one hard switching to HRS or LRS. However, considering the initial status of the memory array, initial weight pattern could be random.

Near exponential relationship exists between the programming voltage amplitude (*V*_*w*_) and synaptic weight change (Δ*w*), also revealed in Fig. 3b. This implies that a small variation in *Vw* can cause a significant deviation in Δ*w*. Therefore, it is essential to estimate the programming efficiency of the proposed CMOS drive circuit. Figure 5b shows the Cadence simulation of the DAC circuitry using 90 nm CMOS technology. A 15.6 mV resolution of the *V*_*DAC*_ for a total 1 V supply is achieved. Figure 5b and Table S1 (Supplementary Information) show overall mapping of spike-timing to DAC code and then to an equivalent voltage (*V*_*w*_ in this case). It has been reported that variation in *V*_*w*_ is less than 5 mV^39,40^. Although a 15.6 mV increase in applied voltage magnitudes even higher than the *V*_READ_ (*i*.*e*., 0.5 V) may not necessarily switch the device, but it is observed that such an increase causes a significant statistical change in Δ*w*.

## Conclusion

In summary, we have presented a CMOS-compatible memristor based on STO_*x*_ exhibiting bipolar resistive switching behavior. First, through electrical and cross-sectional characterizations we have shown that reliable resistive switching in the STO_*x*_ based memristors is attributed to the redox reactions and electronic transport through the localized conductive nano-filamentary pathway. In addition to the exonerated electroforming characteristics, the STO_*x*_ memristors have also shown their scalability potential for future high-density memory applications. Secondly, we have demonstrated a hybrid CMOS-memristor approach to successfully mimic time-dependent, such as *p*- and *t*-STDP, and rate-dependent such as BCM synaptic learning rules. As such, this study is a step towards the realization of an adaptive neuromorphic network by utilizing high order (triplet and quadruplet) learning rules.

## Methods

### Device fabrication

The STO_*x*_ synaptic devices are fabricated as cross-point devices and array in metal-insulator-metal (MIM) configuration with the stack of Pt/Ti/STO_*x*_/Pt/Ti/SiO_2_/Si. Several devices with active areas of 2×2 µm^2^, 4×4 µm^2^, 10×10 µm^2^, 20×20 µm^2^, 40×40 µm^2^, 80×80 µm^2^ and 100×100 µm^2^ are patterned by following standard photolithography and thin film deposition processes. The bottom Pt (15 nm)/Ti (7 nm) electrodes are patterned onto a SiO_2_ (300 nm)/Si substrate by electron beam (e-beam) evaporation. As a switching layer, 25 nm thin film of oxygen-deficient STO is deposited by using radio frequency sputtering (with 100 W power) from a commercial ceramic STO target in a pure argon environment under a pressure of 0.46 Pa and at room temperature. In order to complete the MIM structure, top Pt (30 nm)/Ti (5 nm) electrodes are evaporated by the e-beam evaporation at a base pressure of <6×10^−5^ Pa.

### Electrical characterization

The electrical characterization of the STO_*x*_ synaptic devices is performed under ambient conditions by using a Keysight 2912 A source measure unit and Keithley 4200SCS equipped with remote preamplifiers and 4225 pulse modulation units connected to a micro-probe station. For the resistive switching of STO_*x*_ synaptic devices, bias is applied on the bottom Pt electrodes while keeping the top Pt electrodes grounded. High temperature electrical measurements are performed by using an environmentally isolated Linkum chamber connected with Agilent 2912 A source meter.

### X-ray photoelectron spectroscopy

X-ray photoelectron spectroscopy (XPS) analysis is conducted by using a Thermo Scientific K-Alpha instrument utilizing an aluminum *K*α radiation source (1486.6 eV). The XPS spectra are collected from bare STO oxide thin films, sputtered on SiO_2_/Si substrates. Also, a stoichiometric crystalline STO substrate is used as reference. All spectra are resolved by using the standard Gaussian-Lorentzian function followed by the Shirley background correction.

### Photoluminescence spectroscopy

The photoluminescence (PL) emission spectra are obtained using a Horiba Scientific FluoroMax-4 spectrofluorometer. All spectra are collected at room temperature from as-deposited bare STO_*x*_ thin films sputtered on SiO_2_/Si substrates. A laser source with 325 nm of wavelength is used to excite the sputtered thin films.

### Transmission electron spectroscopy

The transmission electron microscopy (TEM), energy-dispersive X-ray spectroscopy (EDS) and electron energy loss spectroscopic (EELS) analyses are performed on pristine and switching STO_*x*_ MIM devices (at least for 50 cycles and subjected to constant bias stresses) using a JEOL 2100 F scanning transmission electron microscope (STEM) with attached Tridium Gatan image filter with an aperture of 5 mm. The TEM samples are prepared by focused ion beam cuts through the MIM structure by using a FEI Scios DualBeam^TM^ system. Cross-sectional STEM micrographs and EELS spectra are collected using a <1.5 nm beam spot. EELS spectra are collected with a dispersion of 0.3 eV per pixel which allowed simultaneous recording of the titanium *L*_2,3_ (Ti–*L*_2,3_) edge and oxygen K (O–K) edge in the regions of interest and across the MIM cross-sections. A power law fit is adopted for the pre-edge background correction while the influence of nearby peaks and plural scattering are reduced by narrow signal windows.

## Supplementary information

Supplementary information

## Data Availability

All data needed to evaluate the conclusions in the paper are present in the paper and/or Supplementary Materials. Additional data related to this paper may be requested from the authors.
