# Supplementary material for: Time and rate dependent synaptic learning in neuro-mimicking resistive memories
Source: Sci Rep. 2019 Oct 28;9:15404. doi: 10.1038/s41598-019-51700-0 (PMC6817848; doi:10.1038/s41598-019-51700-0)
Supplement: Supplementary file 1 — Supplementary information [file 41598_2019_51700_MOESM1_ESM.pdf]

Supplementary Information

Time and rate dependent synaptic learning in neuro-mimicking resistive memories

T. Ahmed,[1,*] *S. Walia,*[1] *E. L. H. Mayes,*[2] *R. Ramanathan,*[3] *V. Bansal,*[3] *M. Bhaskaran,*[1] *S. Sriram*[1,*] *and O. Kavehei*[1,†,*]

[1]Functional Materials and Microsystems Research Group and Micro Nano Research Facility, RMIT University, Melbourne, VIC 3001, Australia

[2]RMIT Microscopy and Microanalysis Facility, RMIT University, Melbourne, VIC 3001, Australia

[3]Sir Ian Potter NanoBioSensing Facility, NanoBiotechnology Research Laboratory, School of Science, RMIT University, Melbourne, VIC 3001, Australia

†Faculty of Engineering and Information Technology, The University of Sydney, NWS 2006, Australia

* Corresponding authors. E-mail: taimurahmad1@gmail.com, Sharath.sriram@gmail.com, omid.kaveh@gmail.com

Content

S1. **Compositional and electronic analyses of STO_x thin films** 2
S2. **Electrical characterisation of STO_x memristive devices** 5
S3. **Cross-sectional analyses of STO_x devices** 9
S4. **Spike-time conversion to voltage** 11
S5. **Event digitizer and time-to-voltage converter** 14

S1. Compositional and electronic analyses of STO_x thin films

The chemical composition of the sputtered STO_x thin film is analyzed by X-ray photoelectron spectroscopy (XPS). To evaluate the stoichiometry, the core-level elemental spectra of sputtered STO_x thin film are compared with the spectra collected from a reference stoichiometric STO (100) substrate, as shown in **Fig. S1**a-c. The Sr 3*d* and O 1*s* spectra of both samples show similar binding energies which are within the margin of measurement error (±0.1 eV). The Sr 3*d* spectra of stoichiometric STO and STO_x (Fig. S1a) are fitted into two split Sr $3d_{5/2}$ orbital components corresponding to Sr^{2+} binding energies at 132.94 eV and 132.97 eV, respectively.[1, 2] The O 1*s* spectra of stoichiometric STO and STO_x (Fig. S1b) are fitted with two distinct oxygen species. The peaks at binding energies of 529.45 eV (for stoichiometric STO) and 529.47 eV (for sputtered STO_x) correspond to O^{2-} ions,[1] while peaks at higher binding energies are associated with the C–O bonds[1-3] arising from adventitious carbon on the surfaces. The Ti 2*p* spectra of the both stoichiometric STO reference substrate and our STO_x are shown in Fig. S1c. The Ti 2*p* spectrum of the stoichiometric STO suggests that Ti is present in its single oxidation state, *i.e.*, Ti^{4+} with Ti $2p_{3/2}$ peak at 458.37 eV.[2, 4, 5] However, the resolved Ti 2*p* spectrum of the sputtered STO_x thin film show two distinct oxidation states with Ti $2p_{3/2}$ peaks at binding energies of 457.9 eV and 456.2 eV. These correspond to the Ti^{4+} and Ti^{3+} oxidation states, respectively.[1, 3] The relative concentration of Ti^{4+} and Ti^{3+} species in STO_x are calculated to be 70.6% and 29.4%, respectively, by integrating the respective fitted peaks. This indicates that the sputtered STO thin films are oxygen-deficient. This can be associated with the sputtering conditions of STO in a pure argon environment where Ar^+ ion bombardment results in the preferential removal of oxygen atoms and creates inherent oxygen vacancies.[6]

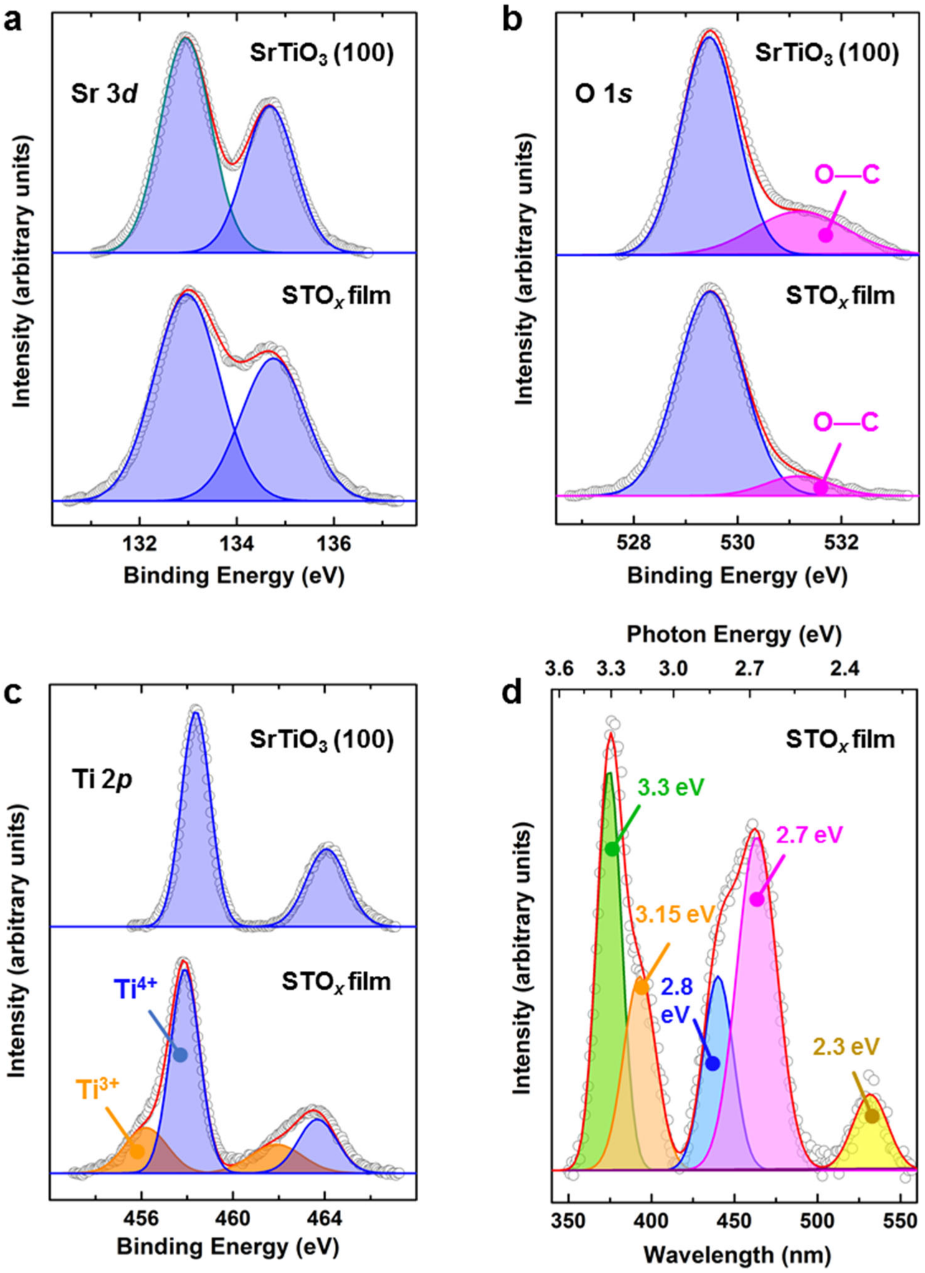

Fig. S1 Material characterization of $SrTiO_3$. The core-level resolved XPS spectra of (a) Sr 3*d* (b) O 1*s* and (c) Ti 2*p* collected from a reference stoichiometric $SrTiO_3$ (100) single crystal and the sputtered STO_x thin film. (d) The resolved photoluminescence emission spectra of STO_x collected at 325 nm excitation wavelength.

To assess the effect of as-grown oxygen vacancies on the electronic structure of STO_x, photoluminescence (PL) spectra are obtained using a 325 nm (3.82 eV) excitation source (Fig. S1d). The PL emission from undoped STO (at room temperature) is associated with the presence of TiO_5 defects.[6-9] Furthermore, it is know that sputter deposition in a pure Ar environment creats as-grown oxygen vacancies (*i.e.*, TiO_5 defects),[9-11] which is also observed in our XPS analysis (explained above). As such, the PL emission is expected from STO_x thin film. Fig. S1d shows the PL spectra collected from bare STO_x thin film at room temperature. The resolved PL spectra show a range of emission components between 2.3 eV and 3.3 eV which can be associated with the recombination of free electrons with in-gap levels and self-trapped exciton recombination.[6, 12] This indicates that as-grown oxygen vacancies introduce in-gap states which result in the overall bandgap reduction of STO_x. As such, the reduced bandgap of STO_x may provide an assistive role in electroforming-free resistive switching behavior at relatively low applied bias than previously reported STO memristors.[13, 14]

S2. Electrical characterisation of STO_x memristive devices

Fig. S2a shows the microscopic photographs of the fabricated STO_x MIM devices with different device area. Fig. S2b shows resistances of the pristine STO_x devices with respect to their active area. The resistances of at least 15 devices, with same active area are measured under a read voltage (V_{READ}) of 0.1 V.

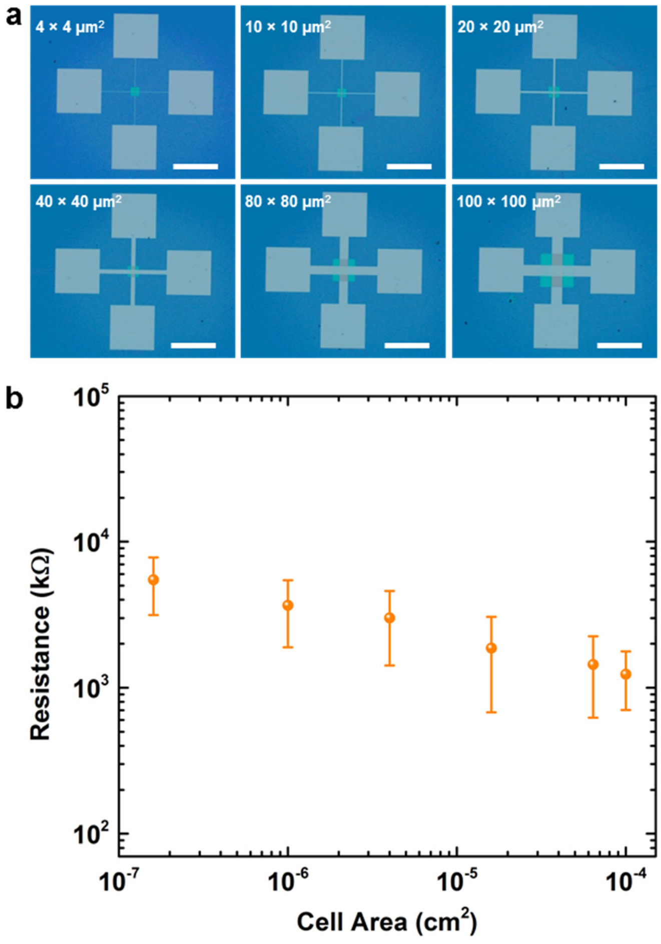

Fig. S2 Electrical characterization of the pristine STO_x devices. (a) Microscopic photographs of the fabricated STO_x devices with different cell areas. Scale bar 400 μm. (b) The pristine resistances measured for at least 15 devices of the same cell area at V_{READ} of 0.1 V. The error bars show the standard deviation in the measurements.

Fig. S3a shows the representative *I–V* characteristics of first SET/REST curves of devices with different cell area. The statistical analysis (Fig. S3b) of V_{SET}/V_{RESET} for at least 15 MIM devices with similar cell area shows that the switching voltages decrease with increasing cell size. This can be associated with the decrease in pristine resistance with increasing cell size as shown in Fig. S2b.

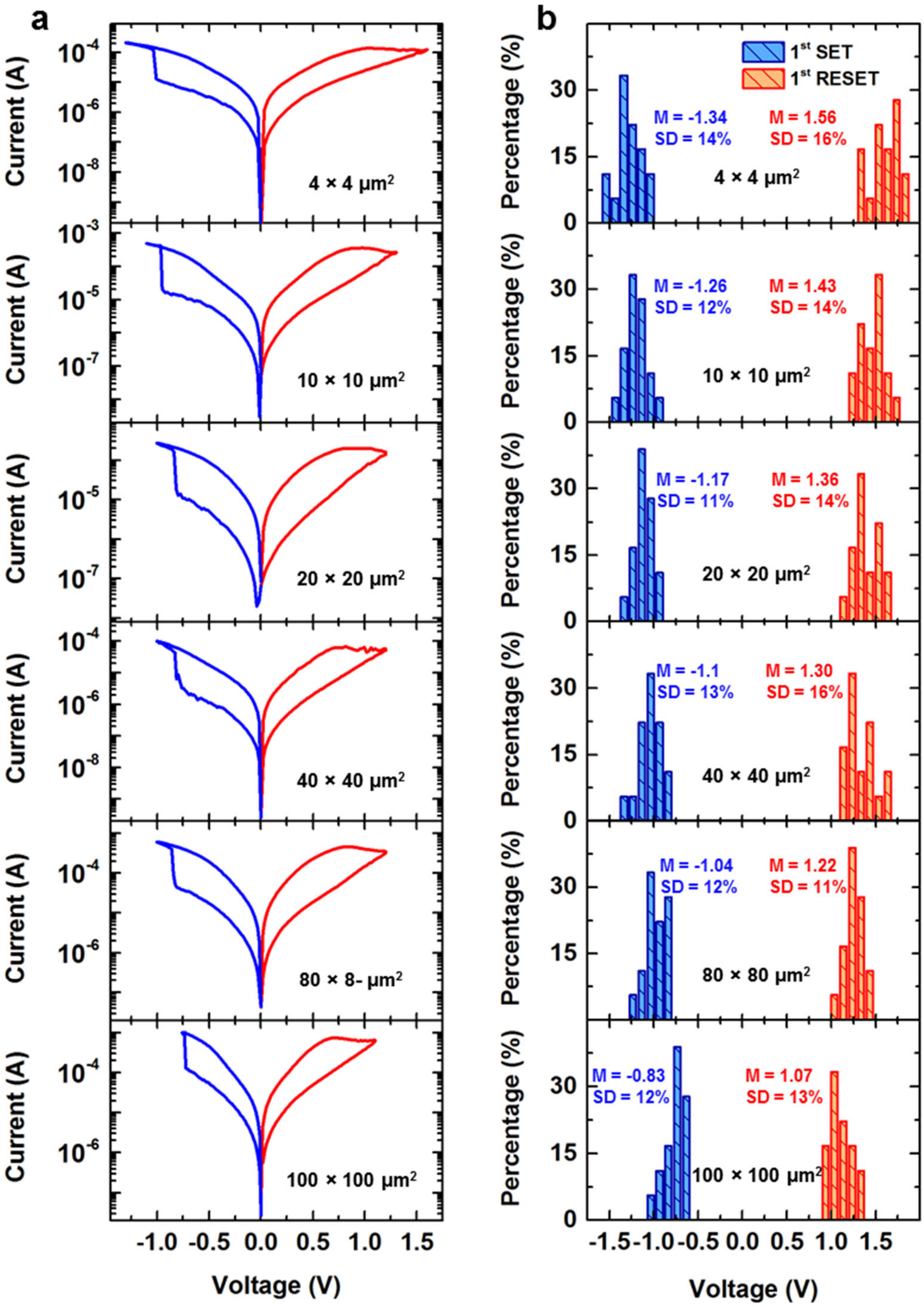

Fig. S3 First SET/RESET characterisation of the STO$_x$ devices. (a) The representative *I*–*V* characteristics of the first SET/RESET sweeps. The current compliance is set at ≥0.5 mA during the *I*–*V* sweeps. (b) The statistics of the first V_{SET} and V_{RESET} of at least 15 MIM devices with same cell sizes (M: mean value and SD: standard deviation).

During the endurance evaluation of STO_x MIM devices, train of WRITE/READ/ERASE/READ voltage pulses are used. **Fig. S4** shows the characterisation of applied voltage pulses.

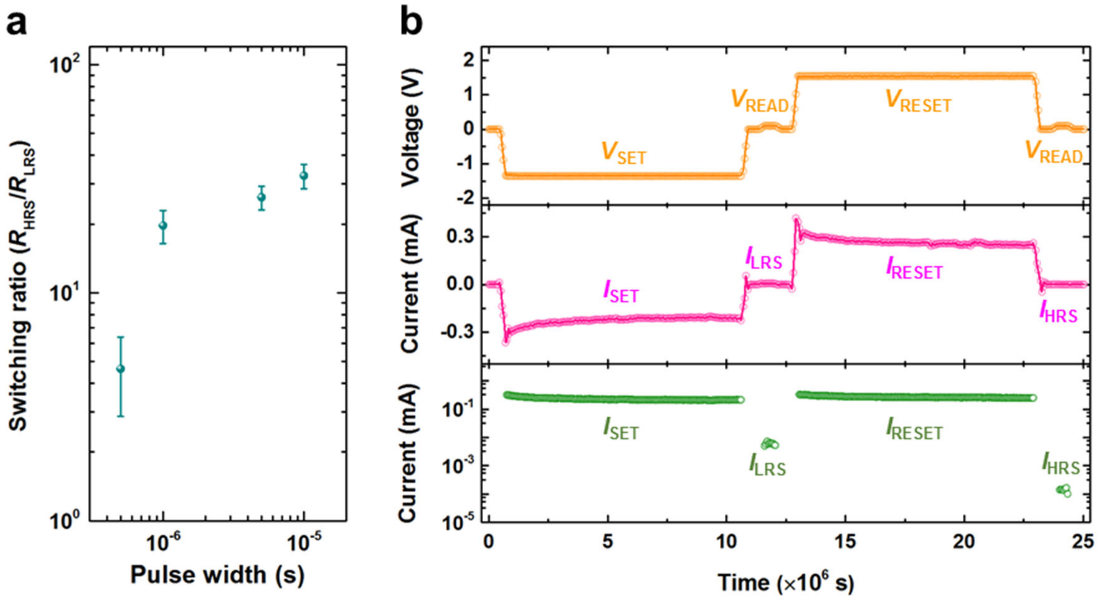

Fig. S4 Characterisation of SET/RESET pulses for the endurance of STO_x MIM devices. (a) Effect of SET and RESET pulse width on switching ratio. The READ pulses with amplitude of 0.1 V and fixed width of 200 ns are used to measure the switching ratios of 15 MIM devices with 10×10 μm^2 active area. (b) Response of the MIM device to the input V_{SET} (-1.4 V)/V_{RESET} (+1.6 V) voltage pulses with 1 μs pulse width (500 ns rise and fall time) and 0.1 V V_{READ} pulses with width of 200 ns. The upper panel shows the input voltage pulses, middle panel shows measured current response to the applied voltage pulses on a linear scale while the lower panel shows only SET/RESET and LRS/HRS read currents on a log scales.

S3. Cross-sectional analyses of STO_x devices

Transmission electron microscope (TEM) and electron energy loss spectroscopy (EELS) techniques are used to analyze the morphology and composition of the STO_x MIM devices. **Fig. S5**a shows a TEM micrograph of the pristine STO_x MIM device. During the TEM and live-FFT observation of the pristine devices, no noticeable crystalline regions are identified in the top Ti or the STO_x functional oxide layer. To assess the electronic composition of the pristine device, the EELS area map and Ti–$L_{2,3}$ and O–K edge profiles are obtained from a line-scan across the MIM structure (Fig. S5b,c respectively). The EELS O–K area map (Fig. S5b) shows the presence of low oxygen content in the STO_x oxide layer which indicates its oxygen deficient stoichiometry. The EELS Ti–$L_{2,3}$ edge profiles collected along a line scan (Fig. S5c) show broad Ti–L_3 and Ti–L_2 peaks at the top Ti/STO_x interface indicate the presence of mixed Ti^{2+} and Ti^{3+} oxidation states.[15] In the functional oxide layer, weak splitting of the t_{2g} and e_g peaks indicate Ti^{4+} oxidation state. However, O–K edge profiles are weak and noisy which makes difficult to clearly distinguish the fine structures and cannot be used to accurately identify the Ti valence.

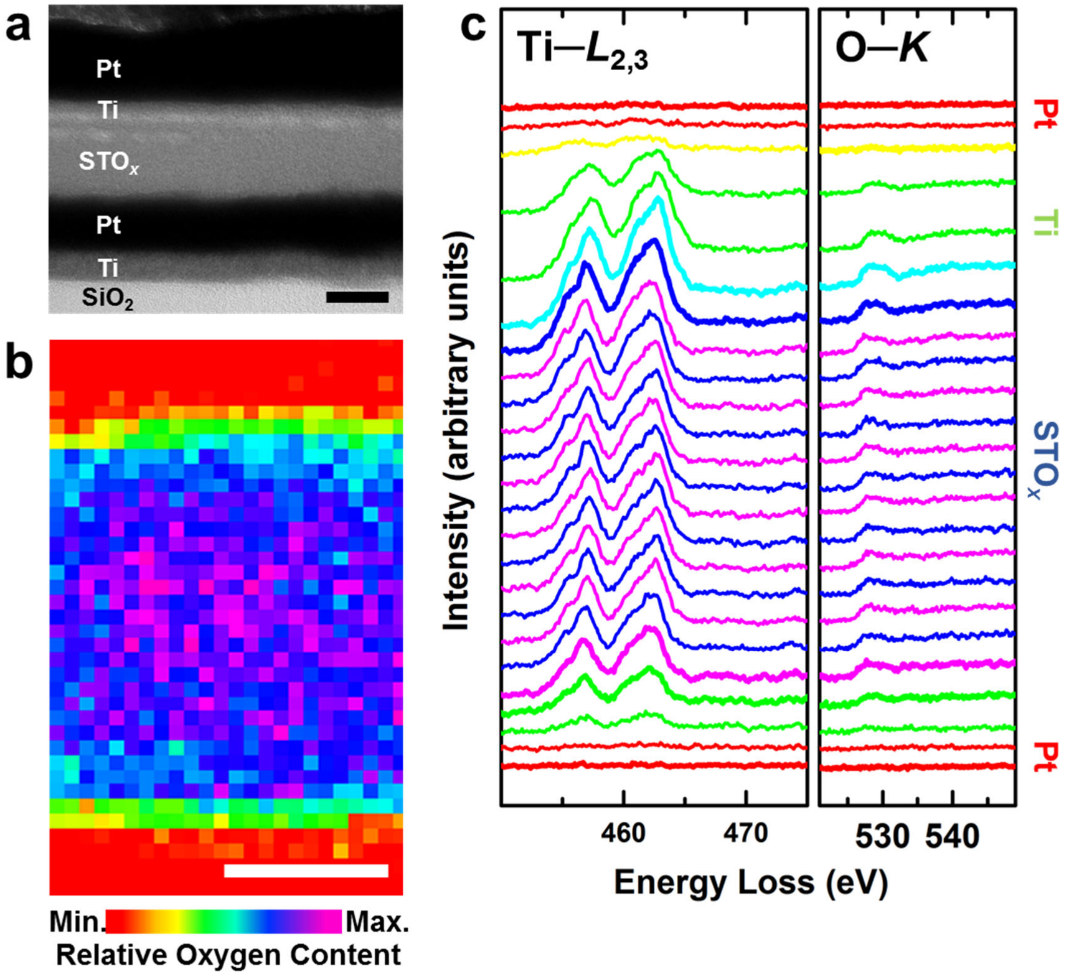

Fig. S5 Microstructure and electronic structure of the pristine STO_x devices. (a) TEM cross-section of a pristine device. Scale bar 20 nm. (b) The EELS O–*K* edge area map of a pristine device. Scale bar 20 nm. (c) The EELS Ti–$L_{2,3}$ and O–*K* edge profiles along a line scan across the pristine device.

S4. Spike-time conversion to voltage

The time-to-digital-to-voltage circuitry converts spike-timing information into corresponding voltage magnitude. The corresponding voltages are applied to the bottom or top electrode of the STO_x synaptic devices, depending on the sign of Δt_1 (potentiation or depression). **Table S1** lists the selected Δt_1 values and corresponding voltage amplitudes.

Table S1. Conversion of the spike-timing information to voltage. Selected values of Δt_1 are simulated to obtain corresponding voltage amplitudes.

Depression		Potentiation			
Δt_1 (ms)	ΔV (V)	Δt_1 (ms)	ΔV (V)		
			Δt_2 = 10 ms	Δt_2 = 80 ms	Δt_2 = 160 ms
-45	0.500	3	-0.800	-0.900	-1.200
-42	0.529	6	-0.778	-0.875	-1.167
-39	0.557	9	-0.757	-0.850	-1.135
-36	0.586	12	-0.735	-0.825	-1.103
-33	0.614	15	-0.714	-0.800	-1.071
-30	0.643	18	-0.692	-0.775	-1.039
-27	0.671	21	-0.671	-0.750	-1.007
-24	0.700	24	-0.650	-0.725	-0.975
-21	0.729	27	-0.628	-0.700	-0.942
-18	0.757	30	-0.607	-0.675	-0.910
-15	0.786	33	-0.585	-0.650	-0.878
-12	0.814	36	-0.564	-0.625	-0.846
-9	0.843	39	-0.542	-0.600	-0.814
-6	0.871	42	-0.521	-0.575	-0.782
-3	0.900	45	-0.500	-0.550	-0.750

Table S2. Comparison of hippocampal data set and STO_x memristors. The synaptic weight change corresponding to different spike time differences is listed for both 2-pre-1-pre and 1-pre-2-post triplet pairing configurations. The hippocampal data set is taken from Ref.[16]

**Triplet pairing**	**Timing difference**		**Weight change**	
	Δt_1	Δt_2	Hippocampal experiments	STO_x memristors
**2-pre-1-post**	5	-5	-0.01±0.04	-0.01±0.02
	10	-10	0.03±0.04	0.01±0.02
	15	-5	0.01±0.03	0.01±0.02
	5	-15	0.24±0.06	0.28±0.03
**1-pre-2-post**	-10	10	0.34±0.04	0.33±0.05
	-5	5	0.33±0.04	0.33±0.05
	-5	15	0.22±0.08	0.23±0.04
	-15	5	0.29±0.05	0.31±0.06

Table S3. Comparison of visual cortex data set and STO_x memristors. The synaptic weight change corresponding to different spike rates is listed for Δt = 10 ms and Δt = -10 ms. The visual cortex data is taken from Ref.[16]

Spike rate ρ (Hz)	Cortex		STO_x memristors	
	Δt = 10 ms	Δt = -10 ms	Δt = 10 ms	Δt = -10 ms
0.1	-0.29±0.08	-0.04±0.05	-0.28±0.03	-0.006±0.02
10	-0.41±0.11	0.14±0.10	-0.43±0.04	0.13±0.03
20	-0.34±0.10	0.29±0.14	-0.35±0.04	0.31±0.05
30	0	0.4±0	0±0.01	0.37±0.04
40	0.56±0.32	0.53±0.11	0.37±0.04	0.37±0.04
50	0.75±0.19	0.56±0.26	0.37±0.04	0.37±0.04

S5. Event digitizer and time-to-voltage converter

The T2D (time-to-digital) module of our proposed CMOS circuit includes a timing control unit and a decoder (shown in **Fig. S6**a). The timing control unit is a fully digital unit that receives pre and post digital spikes and generates timing interval signals solely based on counters. It can be configured for multiple protocol implementation and Δt detection.[17] The resolution of timing detection for any Δt is identified by (1) meaningful changes in synaptic weight (Δw) as the result of applying slight changes in voltage, and (2) resolution of overall time-to-voltage (V_w) conversion which is mainly depends on the DAC resolution. The T2D module passes a multi-bit spike timing (ST) digital signal to a decoder where two multi-bit horizontal/vertical select (HS/VS) digital signals are generated to adjust the number of resistors in series in our resistive DAC. The ST signal contains information about $\Delta t_1$and Δt_2 and their different configurations which then are translated into an equivalent voltage (V_w) to be generated. It also includes flags that are part of Sel_R and Sel_C and identify whether a Δt is positive or negative, hence, applying V_w to the top or the bottom electrodes.

Fig. S6b shows schematic of a single cell with two polysilicon resistors, controls and input/output signals. For a k-bit DAC, 2^k resistors are required. More detail on the implementation of the DAC and voltage follower/buffer (VF) is provided in Ref.[18-20]

Fig. S6c illustrates generation of an internal reference voltage (V_{ref}) by using a voltage divider and also demonstrates a regulated current mirror to generate and regulate a fixed reference current to the chain of DAC resistors. The signal ENA provides the option to minimize the static current flowing through the resistor chain when the time-to-voltage circuit is disabled. It is worth highlighting again that these signals are affecting Sel_R and Sel_C to select a device in the array.

As stated, voltage V_{DAC} identifies maximum required voltage for programming the STO_x memristor. In case of V_{DAC} = 700 mV, 6-bit resistive DAC, and 1.2 V supply voltage in 90 nm standard CMOS technology, a 543 mV dynamic range on V_w, 191 μA active mode current, 99 nA standby mode leakage current at room temperature, and a 235 mV/μs slew rate is achieved.[18] The circuit also demonstrates a strong accuracy of ±5 mV with 8.5 mV step sizes. While we have modified the design, the original design is reported to have an area of around 175×175 μm^2 capable of driving up to 1 mm^2 of digital IP block.[18] We have added a fully digital timing control unit and removed *n*-well bias generation. While the main analog components are still part of the circuit, we estimate an area reduction of at least 20% is achievable in the modified time-to-voltage circuitry in comparison with the original body bias generator circuitry.

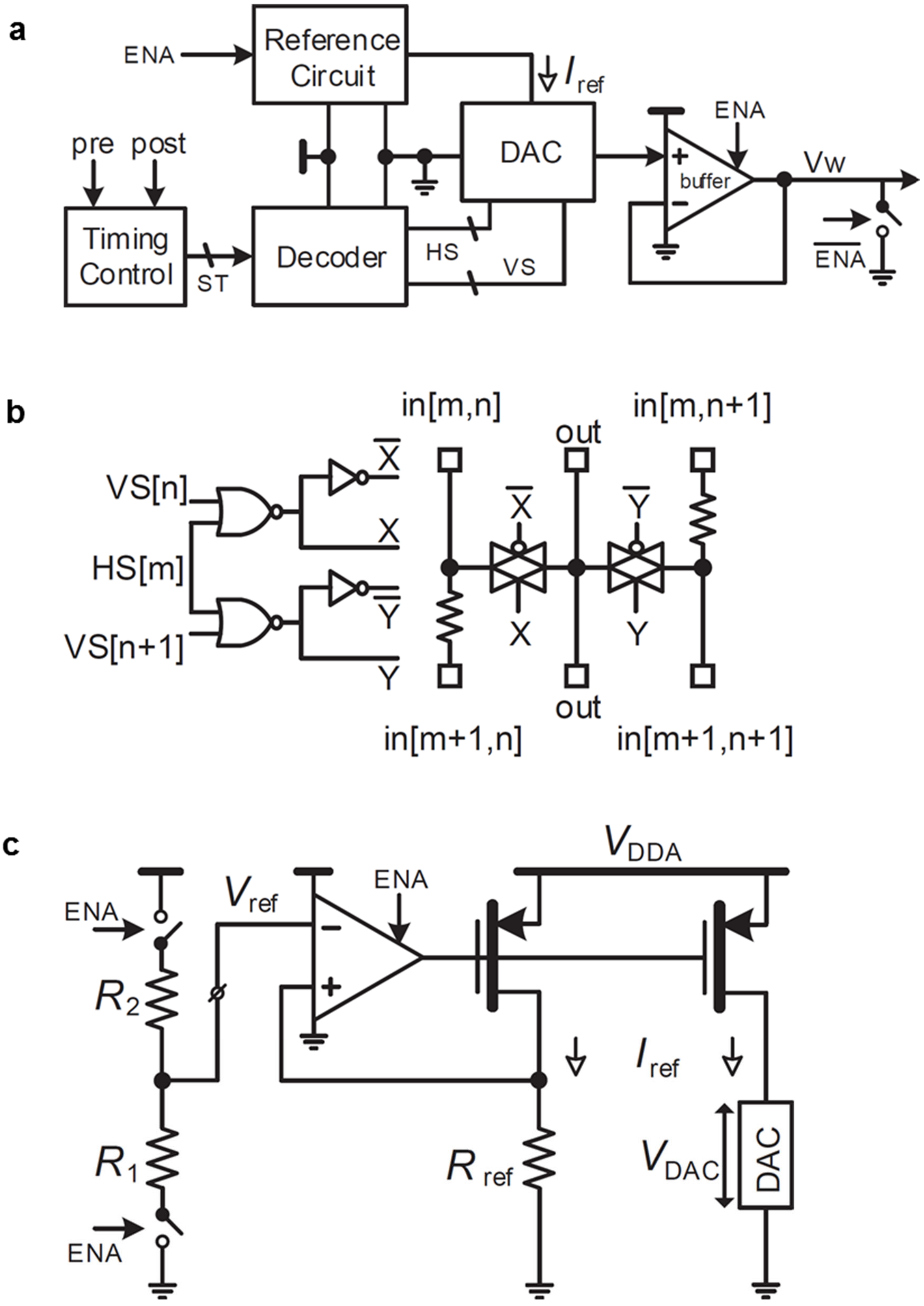

Fig. S6 Event digitization and Time-to-Voltage conversion. Schematics of (a) time-to-digital module, (b) a single cell of resistive DAC and (c) a circuit to generate and regulate reference current and voltage for DAC.

References

1. D. Ehre, H. Cohen, V. Lyahovitskaya and I. Lubomirsky, *Phys. Rev. B*, 2008, **77**, 184106.

2. P. V. Nagarkar, P. C. Searson and F. D. Gealy, *J. Appl. Phys.*, 1991, **69**, 459.

3. H. Nili, S. Walia, A. E. Kandjani, R. Ramanathan, P. Gutruf, T. Ahmed, S. Balendhran, V. Bansal, D. B. Strukov, O. Kavehei, M. Bhaskaran and S. Sriram, *Adv. Func. Mater.*, 2015, **25**, 3172-3182.

4. P. A. W. v. d. Heide, Q. D. Jiang, Y. S. Kim and J. W. Rabalais, *Surf. Sci.*, 2001, **473**, 59-70.

5. J. L. Sullivan, S. O. Saied and I. Bertoti, *Vacuum*, 1991, **42**, 1203-1208.

6. D. Kan, T. Terashima, R. Kanda, A. Masuno, K. Tanaka, S. Chu, H. Kan, A. Ishizumi, Y. Kanemitsu, Y. Shimakawa and M. Takano, *Nat. Mater.*, 2005, **4**, 816-819.

7. E. Orhan, F. M. Pontes, C. D. Pinheiro, T. M. Boschi, E. R. Leite, P. S. Pizani, A. Beltrán, J. Andrés, J. A. Varela and E. Longo, *J. Solid State Chem.*, 2004, **177**, 3879-3885.

8. F. M. Pontes, E. Longo, E. R. Leite, E. J. H. Lee, J. A. Varela, P. S. Pizani, C. E. M. Campos, F. Lanciotti, V. Mastellaro and C. D. Pinheiro, *Mater. Chem.Phys.*, 2003, **77**, 598-602.

9. T. Ahmed, S. Walia, J. Kim, H. Nili, R. Ramanathan, E. L. H. Mayes, D. Lau, O. Kavehei, V. Bansal, M. Bhaskaran and S. Sriram, *Nanoscale*, 2017, **9**, 14690-14702.

10. E. Goldenberg, T. Bayrak, C. Ozgit-Akgun, A. Haider, S. A. Leghari, M. Kumar and N. Biyikli, *Thin Solid Films*, 2015, **590**, 193-199.

11. H. Nili, T. Ahmed, S. Walia, R. Ramanathan, A. E. Kandjani, S. Rubanov, J. Kim, O. Kavehei, V. Bansal, M. Bhaskaran and S. Sriram, *Nanotechnology*, 2016, **27**, 505210.

12. M. L. Crespillo, J. T. Graham, F. Agulló-López, Y. Zhang and W. J. Weber, *J. Phys. D: Appl. Phys.*, 2017, **50**, 155303.

13. C. Baeumer, C. Schmitz, A. Marchewka, D. N. Mueller, R. Valenta, J. Hackl, N. Raab, S. P. Rogers, M. I. Khan, S. Nemsak, M. Shim, S. Menzel, C. M. Schneider, R. Waser and R. Dittmann, *Nat. Comm.*, 2016, **7**, 12398.

14. H. Nili, S. Walia, S. Balendhran, D. B. Strukov, M. Bhaskaran and S. Sriram, *Adv. Func. Mater.*, 2014, **24**, 6741-6750.

15. Y. Li, Q. Wang, M. An, K. Li, N. Wehbe, Q. Zhang, S. Dong and T. Wu, *Adv. Mater. Interf.*, 2016, **3**, 1600201.

16. J. P. Pfister and W. Gerstner, *J. Neurosci.*, 2006, **26**, 9673-9682.

17. S. Friedmann, J. Schemmel, A. Grübl, A. Hartel, M. Hock and K. Meier, *IEEE Trans. Biomed. Circuits Syst.*, 2017, **11**, 128-142.

18. M. Meijer and J. P. d. Gyvez, *IEEE Trans. VLSI Syst.*, 2012, **20**, 42-51.

19. M. Meijer, J. P. d. Gyvez, B. Kup, B. v. Uden, P. Bastiaansen, M. Lammers and M. Vertregt, 2010.

20. M. Blagojević, M. Cochet, B. Keller, P. Flatresse, A. Vladimirescu and B. Nikolić, 2016.
